# Post‐COVID‐19: Designing the new normal in general medicine

**DOI:** 10.1002/jgf2.362

**Published:** 2020-07-29

**Authors:** Takashi Watari, Kiyoshi Shikino, Ayako Shibata, Hiromizu Takahashi, Sou Sakamoto

**Affiliations:** ^1^ Postgraduate Clinical Training Center Shimane University Hospital Shimane Japan; ^2^ Department of General Medicine Chiba University Hospital Chiba Japan; ^3^ Department of Obstetrics & Gynecology Yodogawa Christian Hospital Osaka Japan; ^4^ Faculty of Medicine Juntendo University Tokyo Japan; ^5^ Department of Emergency Medicine Asahi General Hospital Chiba Japan

To the Editor,

The impact of COVID‐19 in the last 6 months (as of June 23, 2020) has been over 900 deaths and over 17 700 confirmed cases in Japan.^1^ The negative effects of COVID‐19 to the economy, medical education, and the medical system are countless. However, they allowed us to reconsider a “new normal” for general physicians and gave us a new turning point.^2^ We discussed that the fundamental change in the post‐COVID‐19 era will be that physical and spatial limitations would be erased by technology. Based on the discussion, we suggest changes to three domains in the medical sector (Table 1). First, this pandemic played a selective role in distinguishing between work that can be replaced with online processes and work for which on‐site presence is vital.^3^ Given that general medicine involves the three aspects of clinical practice, education, and research, we believe that education and research are two fields that can rapidly move online. Online medical education will transcend the boundaries between universities and hospitals; outstanding educational opportunities would become open to everyone, and exceptional educators can be selected. Research for general physicians will cover a wide range of healthcare fields, including clinical research that can be performed in primary care settings, family medicine, public health, medical education, quality and safety, medical informatics, etc.^4^ However, this move from the real to virtual world can create disparity between those who can access and use the Internet effectively and those who cannot. Nevertheless, some work would have to inevitably move online post‐COVID‐19. Second, the gap in learning opportunities close to residences will be resolved; the self‐learning ability online will aid improvement in the ability of doctors.^4^ Learning contents will be freely available through universities and well‐known training hospitals. This will reduce the disparity in postgraduate education levels, which was difficult to access in remote areas. Fusion with technology will promote new general medicine; diagnosis through artificial intelligence, virtual reality training for physical examination, and telemedicine are a few examples.^3^ Third, general physicians may change their self‐criteria when choosing a workplace, thanks to freedom from physical restraints. As a doctor, one can decide where and with whom to live and what kind of work to do. Accordingly, with this change in physical and time restrictions, the criteria for evaluating work for rewards and income should also change. This would involve a radical change in the work for general physicians. For instance, it is highly likely that a general physician will be able to perform additional jobs online. Thus, we propose the “new normal” in general medicine based on general theory from existing literature. In Japan, a new training program for primary care physicians was launched in 2018. In other words, we were already gearing up for a “new normal” in healthcare provision. Therefore, rather than lamenting the inevitable changes, we should be professionals who can respond flexibly to the changes in the near future.

**TABLE 1:** Potential changes to the medical system in the post‐COVID‐19 era

	Pre‐COVID‐19	Post‐COVID‐19
Changes in the job description of a general physician in Japan “Clinical practice, education, research”	Face‐to‐face meetings including outpatient clinics and home visits are the norm. Education, meetings, and conferences are mostly conducted in person. Classes and lectures are provided in real time by the instructor. Clinical clerkships are mainly conducted in real clinical settings. Research is limited to universities and some hospitals, and conducting research has been difficult in remote areas for solo practitioners	Online treatment and prescriptions are applied in clinical practice. Advances in diagnostic aid technologies The online system promotes development of training and research across institutions. Clinical clerkship or training is provided on the go, with the addition of simulations, virtual reality, and online education. Online networks will make it easier to participate in research for solo practitioners working in remote areas
Changes in the way general physicians learn (Continuing Professional Development: CPD)	Learners mostly resort to a local society or training hospital to acquire their knowledge or skills. Basically, workshops and conferences are limited to those who directly participate in them. Role models and mentors of primary care physicians are often only close to the learner. Attendance at conferences requires physical mobility	Learning online, including on‐demand content and live streaming. Sharing of education from a distance: liberating sophisticated conferences on the web for all learners. Role models are now easier to access from a distance. Attendance at conferences is possible online as well
Change in physician's workstyle	Workplace defines where the physician lives. Supplying primary care to remote areas requires physicians to stay there. Wages and compensation are paid in units of hours and days	Possibility of resolving time and space constraints enables diversity of working style. The importance of considering where you live decreases The evaluation parameters for physician's work may change

## CONFLICT OF INTEREST

None.
